# Literary Notes

**Published:** 1908-03

**Authors:** 


### LITERARY NOTES.

*American Medicine,* formerly edited by Dr. George M. Gould as a weekly medical newspaper and later became a monthly, has been removed from Philadelphia to New York. It has put itself into the $1.00 class and appears in its January issue with a new declaration of principles. Dr. Frank Clark Lewis is the managing editor. Its general offices are at 84 William Street, New York, and its publication offices at 189 College Street, Burlington, Vt.

---

THE *Archives of Diagnosis,* a quarterly journal devoted to the study and progress of diagnosis and prognosis, made its first appearance under the date of January, 1908. It is edited by Heinrich Stern, New York. Its subscription price is $1.00 a year.

